# Endothelial glycocalyx degradation in multisystem inflammatory syndrome in children related to COVID-19

**DOI:** 10.1007/s00109-022-02190-7

**Published:** 2022-03-26

**Authors:** Noemi Veraldi, Romain R. Vivès, Géraldine Blanchard-Rohner, Arnaud G. L’Huillier, Noemie Wagner, Marie Rohr, Maurice Beghetti, Ariane De Agostini, Serge Grazioli

**Affiliations:** 1 Department of Diagnostics, Division of Clinical Pathology, Geneva University Hospitals, Geneva, Switzerland; 2 University Grenoble Alpes, CNRS, CEA, IBS, Grenoble, France; 3 Pediatric Immunology and Vaccinology Unit, Geneva University Hospitals and Faculty of Medicine, Geneva, Switzerland; 4 Center of Vaccinology, Geneva University Hospitals, Geneva, Switzerland; 5 Laboratory of Virology, Geneva University Hospitals and Faculty of Medicine, Geneva, Switzerland; 6 Pediatric Infectious Diseases Unit, Geneva University Hospitals and Faculty of Medicine, Geneva, Switzerland; 7 Pediatric Cardiology Unit, Geneva University Hospitals and Faculty of Medicine, Geneva, Switzerland; 8 Department of Pathology of Diagnostics, Geneva University Hospitals and Faculty of Medicine, Geneva, Switzerland; 9 Division of Neonatal and Pediatric Intensive Care, Department of Pediatrics, Gynecology and Obstetrics, Geneva University Hospitals and Faculty of Medicine, 6, rue Willy-Donzé, 1211 Genève 14, Geneva, Switzerland

**Keywords:** Glycocalyx, MIS-C, COVID-19, Inflammation, Endothelium, Heparan sulfate

## Abstract

**Abstract:**

Multisystem inflammatory syndrome in children (MIS-C) represents a rare but severe complication of severe acute respiratory syndrome coronavirus 2 infection affecting children that can lead to myocardial injury and shock. Vascular endothelial dysfunction has been suggested to be a common complicating factor in patients with coronavirus disease 2019 (COVID-19). This study aims to characterize endothelial glycocalyx degradation in children admitted with MIS-C. We collected blood and urine samples and measured proinflammatory cytokines, myocardial injury markers, and endothelial glycocalyx markers in 17 children admitted with MIS-C, ten of which presented with inflammatory shock requiring intensive care admission and hemodynamic support with vasopressors. All MIS-C patients presented signs of glycocalyx deterioration with elevated levels of syndecan-1 in blood and both heparan sulfate and chondroitin sulfate in the urine. The degree of glycocalyx shedding correlated with tumor necrosis factor-α concentration. Five healthy age-matched children served as controls. Patients with MIS-C presented severe alteration of the endothelial glycocalyx that was associated with disease severity. Future studies should clarify if glycocalyx biomarkers could effectively be predictive indicators for the development of complications in adult patients with severe COVID-19 and children with MIS-C.

****Key messages**:**

- Children admitted with MIS-C presented signs of endothelial glycocalyx injury with elevated syndecan-1 and heparan sulfate level.
- Syndecan-1 levels were associated with MIS-C severity and correlated TNF-α concentration.
- Syndecan-1 and heparan sulfate may represent potential biomarkers for patients with severe COVID-19 or MIS-C.

**Supplementary information:**

The online version contains supplementary material available at 10.1007/s00109-022-02190-7.

## Introduction

The coronavirus disease (COVID-19) pandemic caused by severe acute respiratory syndrome coronavirus 2 (SARS-CoV-2) represents one of the most prominent global health threat of this century, with more than 350 million infected individuals and 5.5 million deaths worldwide [1]. While adults have suffered the highest rates of morbidity and mortality related to COVID-19, children were thought to be mostly spared, with 90% being either asymptomatic or mildly affected [2]. Reports of children presenting with severe inflammatory shock and multiple organ dysfunction mimicking both Kawasaki Disease and Toxic Shock Syndrome [3–6] emerged in the later phase of the first wave. This new syndrome was later referred as Multisystem Inflammatory Syndrome in Children (MIS-C) [7, 8] or pediatric multisystem inflammatory syndrome [9]. While the pathophysiology of MIS-C remains largely unknown, current evidence from clinical studies suggests a post-infectious process characterized by a dysregulated immune response in genetically and immunologically susceptible hosts [10–15]. A common feature of children presenting with MIS-C is the involvement of the cardiovascular system, with elevated myocardial injury markers, N-terminal probrain natriuretic peptide (NT-proBNP) and cardiac troponin, and abnormal echocardiographic findings with left ventricular systolic and diastolic dysfunction and coronary artery abnormalities [3].

The endothelial glycocalyx (from now on simply referred to as “glycocalyx”), which covers the luminal surface of endothelial cells, plays a key role for the maintenance of vascular homeostasis, including, but not limited to, the negative charges provided by the glycosaminoglycan chains of proteoglycans, e.g., heparan sulfate and chondroitin sulfate, which form a shield around cells but also provide sites for multiple interactions. If the glycocalyx is damaged, glycocalyx-shedding products can be measured in the plasma, typically syndecan-1, proteoglycan, and heparan sulfate. Several studies have shown that the amount of glycocalyx-shedding products correlates with the severity of ischemia/reperfusion injury in patients after major vascular surgery or acute myocardial infarction [16, 17].

Damage to the glycocalyx has been reported in trauma and septic patients [18–20] but also lately in adult patients with severe COVID-19 [21–25]. Recent reports and commentaries have suggested that a dysregulated endothelium might play a central role in the pathogenesis of acute respiratory distress syndrome (ARDS) and multiple organ failure in COVID-19. Interestingly, a recent study demonstrated the presence of autoantibodies against endothelial and cardiac tissue in MIS-C patients suggesting that damage to the endothelium may also play a role in the pathogenesis of MIS-C [26].

The overall aim of this hypothesis-generating study was to characterize glycocalyx alterations in children admitted with MIS-C. Our specific objectives were as follows: (1) to compare plasma and urinary glycocalyx markers in MIS-C patients presenting with shock to MIS-C patients without shock and healthy volunteers; (2) to determine the correlation of syndecan-1 levels and myocardial injury and proinflammatory markers.

## Materials and methods

### Study participants

Between April 1, 2020, and April 2, 2021, we prospectively enrolled all children (0–16 years old) admitted to the Geneva University Hospitals with a diagnosis of MIS-C according to World Health Organization (WHO) case definition. Real-time polymerase chain reaction (RT-PCR) for SARS-CoV-2 and SARS-CoV-2 immunoglobulin A (IgA) and immunoglobulin G (IgG) serologies were performed according to Grazioli et al. [27]. Demographic, laboratory, and physiological variables were documented for each participant at hospital admission and during hospital stay. Detailed patient characteristics at study inclusion as well as a description of the subsequent clinical course for a subset of the patients enrolled in the present study were recently published in two case series [27, 28]. MIS-C patients were categorized into MIS-C with shock or without shock as per Goldstein 2005 definition of cardiovascular failure [29]. The number of organ dysfunction was evaluated using the modified pediatric logistic organ dysfunction (PELOD)-2 score [30]. Five healthy age-matched children served as controls. All blood and urine samples were collected as part of standard clinical care. Immediately after centrifugation, plasma or serum aliquots were frozen and kept at − 80 °C. Urinary creatinine analysis was performed as standard routine. Clinical laboratory test values at the time of hospital admission and during hospital stay were included in our analysis, when available.

### Circulating markers of glycocalyx dysfunction, inflammation, and myocardial injury

Plasma or serum levels of syndecan-1 were measured using commercially available enzyme-linked immunosorbent assay kits (human anti-CD138 ELISA kit, Diaclone, France) according to the manufacturer’s instruction. Urine samples were filtered (0.22 µm, Millipore, USA) and kept frozen at − 20 °C. Centrifugation at 2000 rpm for 10 min at 4 °C was performed on thawed samples (Eppendorf model 5804, USA) to eliminate possible cryoprecipitates before the isolation of glycosaminoglycans. Heparan sulfate and chondroitin sulfate were purified from urine by diethylaminoethanol-sephadex (GE Healthcare) and concentrated desalted fractions were analyzed by enzymatic depolymerization and reverse-phase ion-pairing high-performance liquid chromatography [31]. Troponin-T, D-dimer, NT-proBNP, albumin, tumor necrosis factor α (TNF-α), and interleukin-6 (IL-6) were measured as part of clinical routine at admission and then repeatedly, according to clinical evolution in the laboratory of the Geneva University Hospitals as previously described [27].

### Statistical methods

Data are presented as absolute numbers, percentages, and medians with interquartile range (IQR). Continuous variables were compared using either the Mann–Whitney *U* test or the Kruskal–Wallis test, as appropriate, and categorical variables were compared using Fisher exact and chi-square tests. To correct for multiple testing in comparisons of syndecan-1 levels per participant group, we used the false discovery rate (FDR) approach of Benjamini, Krieger, and Yekutieli, setting a *q*-value < 0.05 as significant. Two-way repeated analysis of variance (ANOVA) with Tukey post hoc *t-*test was performed to determine difference in heparan sulfate and chondroitin sulfate disaccharides between healthy controls, non-shock, and shock MIS-C patients. Correlations between syndecan-1 and biological markers or outcome measurements were tested using Spearman rank correlation test. All the tests were two-sided and *P* values less than 0.05 were considered statistically significant. SPSS version 24 (IBM Corporation, Armonk, NY, USA) and GraphPad Prism version 9 (GraphPad Prism Software Inc., San Diego, CA, USA) were used for statistical analyses and preparation of figures.

## Results

During the study period, 17 children/adolescents were admitted to the hospital with a diagnosis of MIS-C. Ten patients presented with shock requiring pediatric intensive care unit (PICU) admission and hemodynamic support with vasopressors. Demographic and clinical characteristics of the patients are summarized in Table 1 and Supplemental Table S1. Of the patients, 88% were male with a median age of 11 years. In total, 29% of MIS-C patients were considered obese based on the WHO definition and children presenting with shock had higher median body mass index as compared to children without shock, which did not reach statistical significance (14.9 [14.1–18.8] vs 20.3 [16.7–26.9]; *P* = 0.064). As expected, when compared to controls, all patients presented in hyperinflammatory state characterized by elevated C-reactive protein (CRP), procalcitonin, IL-6, and TNF-α (Table 2). Proinflammatory markers procalcitonin, IL-6, and TNF-α but not CRP were significantly more elevated in MIS-C patients with shock as compared to MIS-C patient without shock. Furthermore, as compared to MIS-C patients without shock, MIS-C patients presenting with shock had lower platelet counts, higher d-dimers concentration, and lower albumin levels at admission. None of the 17 MIS-C patients met the International Society on Thrombosis and Haemostasis diagnostic criteria for disseminated intravascular coagulation (≥ 5 points) [32] but MIS-C patients with shock presented higher median scores as compared to MIS-C patient without shock (3 [3–3.25] vs 2 [2]; *P* = 0.001).

**Table 1:** Admission characteristics of patients reported with multisystem inflammatory syndrome in children (MIS-C)

Characteristic	MIS-C (total) (*n* = 17)	Non-shock MIS-C (*n* = 7)	Shock MIS-C (*n* = 10)	*P* value^a^
**Age**	11 (8–13)	13 (8–13)	11 (8–13)	.552
**Male**	15 (88.2)	7 (100)	8 (80)	.505
**BMI**	18.3 (14.8–24.9)	14.9 (14.1–18.6)	20.3 (16.7–27)	.064
**Comorbidities**	0	0	0	
**Day of illness at admission**	6 (5–8)	6 (4–8)	6 (5–7)	.764
**Symptoms on presentation**				
Fever	17 (100)	7 (100)	10 (100)	
Respiratory symptoms	12 (70.6)	3 (42.9)	9 (90)	.1
Gastrointestinal manifestations	15 (88.2)	6 (85.7)	9 (90)	1
Mucocutaneous symptoms	12 (70.6)	4 (57.1)	8 (80)	.593
Neurological symptoms	8 (47.1)	2 (28.6)	6 (60)	.335
**Echocardiography**				
LVEF < 55%	6 (35.3)	2 (28.6)	4 (40)	1
Coronary abnormalities	3 (17.6)	1 (14.3)	2 (20)	1
**Interventions**				
ICU admission	12	3	9	
Nb of organ dysfunction	2 (0–3.5)	0 (0–1)	3 (2.8–5)	** < .001**
Invasive mechanical ventilation	2 (11.8)	0	2 (20)	.485
Vasopressors	9 (64)	0	9 (100)	
Fluid bolus at ER (ml/kg)	10 (0–25)	0	21 (14–34)	**.002**
Dialysis	1 (5.9)	0	1 (10)	1.0

Data are presented as median (interquartile range) for continuous variables or no (%) for binary variables

*BMI* body mass index, *ER* emergency room, *LVEF* left ventricular ejection fraction

^a^Non-shock MIS-C vs shock MIS-C, Mann–Whitney *U* test, or Fisher exact test

**Table 2:** Laboratory data of patients reported with multisystem inflammatory syndrome in children (MIS-C)

Laboratory values	MIS-C (total) (*n* = 17)	Non-shock MIS-C (*n* = 7)	Shock MIS-C (*n* = 10)	*P* value^a^
WBC count (× 10^9^/L)	7.9 (6.5–10.35)	8.1 (7.2–8.7)	7.6 (5.6–13)	.845
Neutrophil count (× 10^9^/L)	6.9 (4.65–10.1)	6.9 (4.4–8)	6.8 (4.7–10.7)	.625
Lymphocyte count (× 10^9^/L)	0.6 (0.2–1.2)	0.74 (0.5–2.2)	0.52 (0.16–0.83)	.118
Platelets (× 10^9^/L)	145 (113.5–207.5)	199 (151–235)	127 (100–146.8)	**.019**
CRP (mg/mL)	214(181–286)	198 (136–265)	233 (190–329)	.143
PCT(μg/L)	8 (1.7–19.3)	1.8 (1.1–5.5)	13.9 (7.4–43.9)	**.006**
Il-6 (pg/mL)	93.5 (34.2–229.4)	74.6 (3.9–94.4)	129.5 (88.8–928)	.**03**
TNF-α (pg/mL)	19.8 (11.2–28.7)	11 (4.7–13.9)	27.7 (20.4–43.2)	**.001**
INR	1.08 (1–1.17)	1.06 (1–1.25)	1.09 (1.04–1.16)	.660
PT (%)	86 (72.5–97.5)	80 (62–100)	85 (74.3–92.5)	.590
PTT (sec)	36.9 (31.9–40)	35.3 (31–37)	38.3 (34.1–41)	.241
Fibrinogen (g/L)	5.3 (5–7.4)	5.6 (5.1–8.1)	5.1 (4.5–6.5)	.101
D-dimers (g/L)	3924 (2071–7539)	1969 (1527–2677)	6433 (3908–9178)	.**002**
Albumin (g/L)	30 (26–33)	32 (30–37)	27 (25–31)	.**028**
NT-proBNP (ng/mL)^b^	5422 (2591–21,204)	2722 (1334–3978)	15,724 (4217–32,485)	**.02**
Troponin (ng/mL)	65 (31–214)	133 (35–199)	51 (27–246)	.696

Data are presented as median (interquartile range) for continuous variables or no (%) for binary variables

*CRP* C-reactive protein, *PCT* procalcitonin, IL*-6* interleukin-6, *TNF-α* tumor necrosis factor α, *NT-proBNP* N-terminal probrain natriuretic peptide, *PT* prothrombin time (percentage activity), *PTT* partial thormoboplastin time, *INR* international normalized ratio

^a^Non-shock MIS-C vs shock MIS-C, Mann–Whitney *U* test, or Fisher exact test

^b^Missing NT-proBNP data in 2 patients in the non-shock MIS-C group and 1 patient in the shock-MIS-C group

All MIS-C patients presented signs of myocardial injury with elevated troponin and NT-proBNP levels, with significantly higher NT-proBNP levels in MIS-C patients with shock as compared to MIS-C patients without shock. Echocardiographic examinations were abnormal in 6 patients, with a decreased left ventricular ejection fraction in two patients from the non-shock group and 4 patients from the shock group (Table 1). In addition, one patient from the non-shock group and two patients from the shock group presented coronary artery dilation.

Treatment with intravenous immunoglobulin (IVIG) and corticosteroids were given to 82% of the patients without difference according to the mode of presentation with shock or without shock (Table 3). Six patients received immunomodulatory treatments for persistent fever and worsening of clinical picture with three patients receiving Anakinra, two patients receiving Tocilizumab, and one patient receiving both Anakinra and Tocilizumab. All of the children were treated at admission with broad spectrum antibiotics that could be discontinued at 48 h for negative blood cultures. No patients developed secondary bacterial or viral infection despite the different immunomodulatory treatments they received. Because of an increased risk for thrombosis, all MIS-C patients received prophylactic anticoagulation with either unfractionated heparin (UFH) (at a dose of 10 UI/kg/h intravenous) or low molecular weight heparin (LMWH) (such as enoxaparin at a dose of 0.5 mg/kg/dose, maximal dose of 40 mg subcutaneous q 24 h) during their hospital stay until discharge.

**Table 3:** Treatment and outcome of patients reported with multisystem inflammatory syndrome in children (MIS-C)

Characteristic	MIS-C (total) (*n* = 17)	Non-shock MIS-C (*n* = 7)	Shock MIS-C (*n* = 10)	*P* value^a^
**Treatment**				
IVIG	14 (82.4)	5 (71.4)	9 (90)	.537
Corticosteroids	14 (82.4)	5 (71.4)	9 (90)	.537
Biologics	6 (35.3)	1 (14.3)	5 (50)	.304
**Outcome**				
ICU length of stay (days),	5 (2–7)	NA	7 (4–10)	
Hospital length of stay (days)	7 (7–10)	6 (5–7)	10 (7–14)	**.008**
Survival to discharge	14 (100)	5 (100)	9 (100)	

Data are presented as median (interquartile range) for continuous variables or No (%) for binary variables

*ICU* intensive care unit, *IVIG* intravenous immunoglobulin

^a^Non-shock MIS-C vs shock MIS-C, Mann–Whitney *U* test, or Fisher exact test

All MIS-C patients survived to discharge and had a median hospital length of stay of 7 days, with MIS-C patients with shock staying longer as compared to MIS-C patients without shock.

**Fig. 1:**
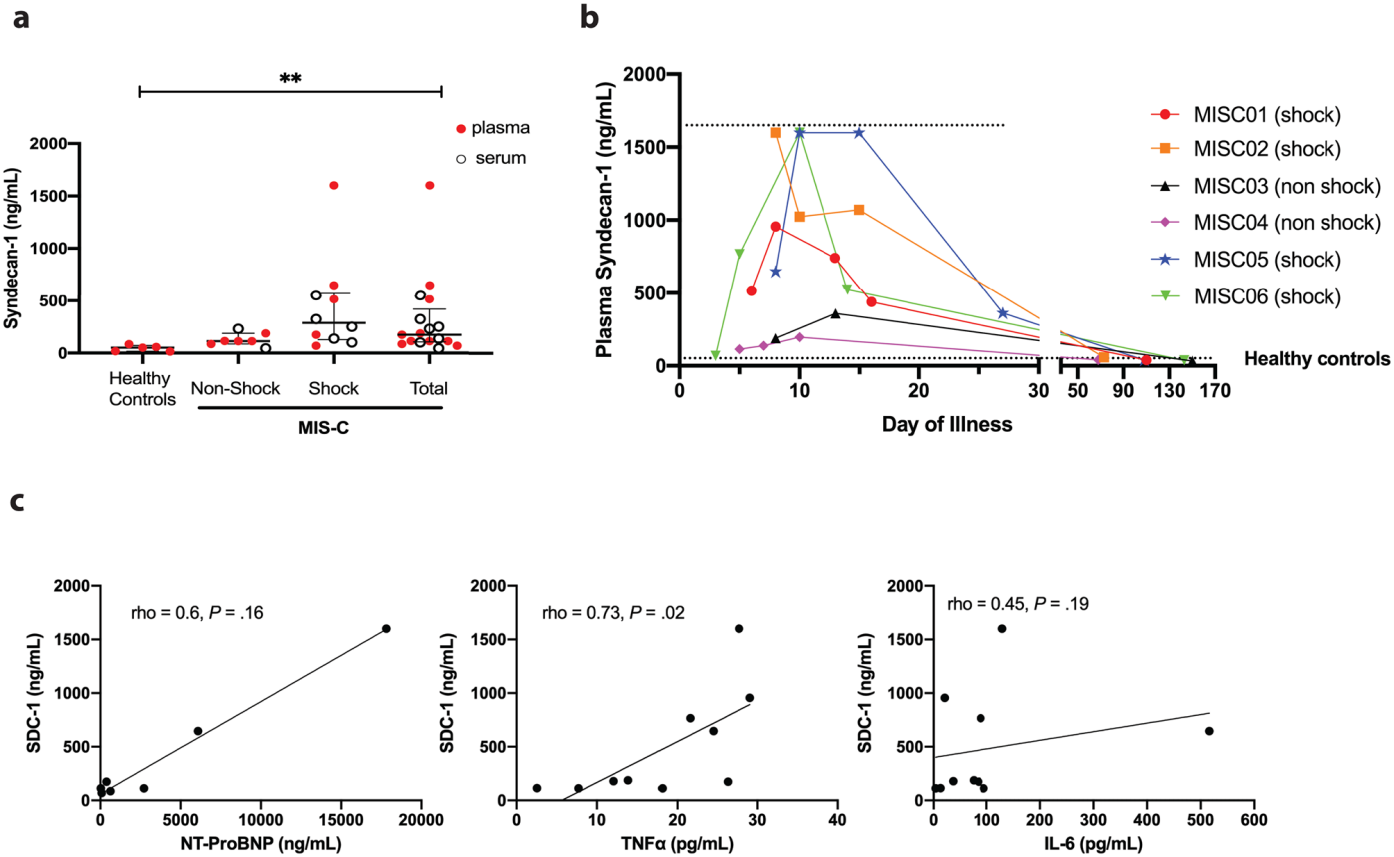
Syndecan-1 concentration increases in children with MIS-C. **a** Admission level of syndecan-1 (ng/ml) in healthy, MIS-C without shock, and MIS-C with shock patients. Data represent median with interquartile range of plasma and serum samples. **P < 0.01, Mann–Whitney test (non-shock MIS-C vs shock-MIS-C), and (healthy controls vs total MIS-C patients). Black open circle = serum sample and red full circle = plasma sample. **b** Time course of syndecan-1 plasma concentration in children admitted with MIS-C since hospital admission. The limit of detection of the ELISA is represented by a dotted line. Control data are the average of three control samples. **c** Correlation of plasma syndecan-1 with NT-proBNP, IL-6, and TNF-α. Spearman correlation test. IL-6, interleukin-6; MIS-C, multisystem inflammatory syndrome in children; NT-proBNP, N-terminal probrain natriuretic peptide; TNF-α, tumor necrosis factor α

Syndecan-1 concentration measured at admission is illustrated in Fig. 1. Because of the limited availability of plasma samples, syndecan-1 was measured in either plasma or serum (MISC07–MISC14). Preliminary data comparing syndecan-1 concentration measured in plasma and serum samples from healthy volunteers showed a lower median syndecan-1 concentration in serum samples as compared to plasma samples (11.7 [7.3–14.4] vs 16.8 [11.9–26.3]; *P* = 0.0625). Therefore, to avoid the confounding effect of the type of sample (serum vs plasma), we compared syndecan-1 concentration at admission only on plasma samples. We found that syndecan-1 median (IQR) concentration at admission was significantly elevated in all MIS-C patients as compared to healthy matched controls (144 [105–547] vs 50 [15–71] ng/mL; *P* = 0.001), with higher levels in MIS-C patients with shock as compared to MIS-C patients without shock, without reaching statistical significance (514 [122–1123] vs 113 [99–151] ng/mL; *P* = 0.2). Furthermore, to correct for the different response and to normalize results, syndecan-1 levels were also expressed as ratio on the relative healthy control (serum or plasma) used in each test (Supplemental Fig. S1a). Accordingly, higher syndecan-1 ratios in shock patients compared to non-shock patients (14.5 [6.6–21.7] vs 2.6 [2.2–4.0]; *P* = 0.0012) were observed. Interestingly, the syndecan-1 value of patient MISC06 at admission was similar to levels of healthy patients (68.6 ng/mL, ratio 1.3) but increased to shock levels the following day (767 ng/mL, ratio 13.8). There was no correlation between admission syndecan-1 levels and hospital length of stay (*R* = 0.389 and *P* = 0.122) but a trend toward a correlation between syndecan-1 levels and the number of organ dysfunction (*R* = 0.481 and *P* = 0.0523).

For some patients, it was possible to monitor syndecan-1 during the hospitalization until complete recovery. Syndecan-1 increased after admission, peaked around day 10–15 of illness with four patients presenting levels above 1000 ng/mL, and returned to normal value in all monitored patients at a median time (IQR) of 109 days (72–145 days) following symptom onset (Fig. 1b and Supplemental Fig. S1b, c).

**Fig. 2:**
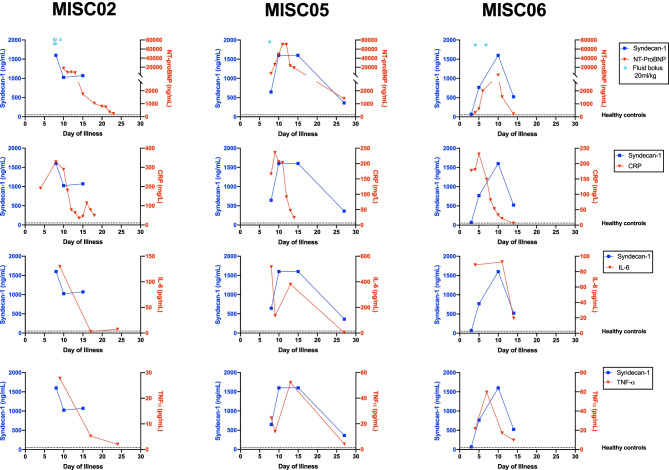
Time course of syndecan-1 concentration according to NT-proBNP, CRP, IL-6, and TNF-α plasma concentration in three MIS-C patients with shock. Control data are the mean of three control samples. CRP, c-reactive protein; IL-6, interleukin-6; MIS-C, multisystem inflammatory syndrome in children; NT-proBNP, N-terminal probrain natriuretic peptide; TNF-α, tumor necrosis factor α

Because of the temporal relationship between the increase in inflammatory mediators, NT-proBNP, and syndecan-1, we explored the correlation between syndecan-1 plasma levels and those biomarkers (Fig. 1c) at admission and demonstrated their time course in three MIS-C patients presenting with shock (Fig. 2). Although NT-proBNP and syndecan-1 levels appeared to follow a similar time course over time, there was no significant correlation between those two markers at admission (rho = 0.60, *P* = 0.166). Among inflammatory cytokines, only TNF-α was positively correlated with syndecan-1 (rho = 0.73, *P* = 0.02). Of note, in all three patients, CRP levels started to decrease early, while NT-proBNP, syndecan-1, and cytokine concentration were still at their peak level.

To further investigate glycocalyx degradation in MIS-C patients, urinary levels of heparan sulfate and chondroitin sulfate and their structural composition were measured by reverse-phase ion-pairing high-performance liquid chromatography analysis after digestion of isolated glycosaminoglycans into constituent disaccharides. The analysis was performed on those patients for which urine samples were available. A significant higher median concentration of heparan sulfate and chondroitin sulfate was detected in urine samples from MIS-C patients as compared to healthy controls (Table 4). Despite being collected at different time point of illness, urinary levels of both glycosaminoglycans were more elevated in MIS-C patients presenting with shock as compared to non-shock patients without reaching statistical significance. Since all patients were treated with either UFH or LMWH, the potential presence of heparin in the urine could have affected urinary heparan sulfate quantification. However, the patients received only prophylactic dose of LMWH or UFH with activated partial thromboplastin time (aPTT) remaining under the therapeutic range (46–79 s) [33] indicating low level of heparin activity. Furthermore, given enoxaparin’s half-life of 5–7 h with an anticoagulant Xa effect of nearly 12 h [34], we did not expect to find enoxaparin in urine samples collected 12 h after subcutaneous administration. Moreover, to exclude the possible interference of enoxaparin treatment with urinary levels of heparan sulfate, we compared the concentration of heparan sulfate measured immediately before (MISC16) or 12 h after the administration of enoxaparin (MISC17) in patients from the same category and found no significant difference (Table 4 and Supplemental Fig. S2). Since traces of UFH might have been present in shock patients under continuous heparin infusion, namely MISC05, we subtracted the contribution on heparin’s main disaccharide NS2S6S and recalculated the content of the other disaccharides accordingly. Corrected data of heparan sulfate for patient MISC05 were still in the range of shock patients, higher than both non-shock and control subjects (Supplemental Fig. S3).

**Table 4:** Urinary heparan sulfate (HS) and chondroitin sulfate (CS) isolated from patients with multisystem inflammatory syndrome in children (MIS-C)

Sample	Day of illness	μg HS	μg HS/mg creatinine	μg CS	μg CS/mg creatinine
**Non-shock MIS-C**					
MISC15	6	8.7	1.5	34.5	5.8
MISC16	4	5.8	2.4	44.2	18.2
MISC17	7	7.9	1.4	34.3	6.2
Median (IQR)		7.9 (5.8–8.7)	1.5 (1.4–2.4)	34.5 (34.3–44.2)	6.2 (5.8–18.2)
**Shock MIS-C**					
MISC05	11	11.3	6	47.7	24.6
MISC02	23	7.7	4.8	17.3	10.8
MISC18	7	48.7	5.7	116.4	13.6
Median (IQR)		11.3 (7.7–48.7)	5.7 (4.8–6)	47.7 (17.3–116.4)	13.6 (10.8–24.6)
Median (IQR) All MIS-C		8.3 (7.2–20.7)	^*^3.6 (1.5–5.8)	39.4 (30.1–64.9)	^*^12.2 (6.1–19.8)
**Healthy controls**					
CTRL1	-	3.9	0.4	14.1	1.4
CTRL2	-	6.9	0.7	17.1	1.7
CTRL3	-	4.6	0.5	11.9	1.2
Median (IQR)		4.6 (3.9–6.9)	^*^0.5 (0.4–0.7)	14.1 (11.9–17.1)	^*^1.4 (1.2–1.7)

^*^P < .05, Mann–Whitney test median heparan sulfate from all MIS-C patients vs median heparan sulfate from healthy controls and median chondroitin sulfate from all MIS-C patients vs median chondroitin sulfate from healthy controls

**Fig. 3:**
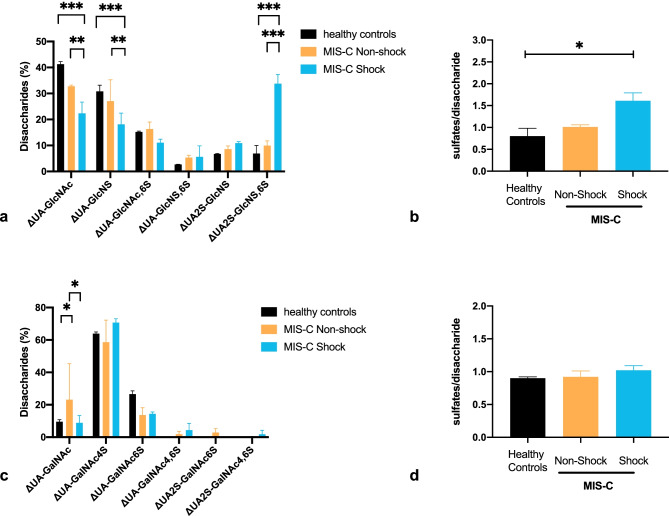
Sulfation pattern of urinary heparan sulfate and chondroitin sulfate from children with MIS-C. The percentage of variously substituted disaccharides is reported for HS in **a** and for CS in **c**. The overall sulfation degree (sulfates/disaccharide) is shown for HS in **b** and for CS in **d**. Data represent median with interquartile range. *P < 0.05, **P < 0.01, ***P < 0.001, two-way analysis of variance (ANOVA) with Tukey post hoc *t*-test (**a** and **c**); *P < 0.05, Kruskal–Wallis test with FDR correction (**b** and **d**). ΔUA, unsaturated uronic acid ± 2-O-sulfation (2S); GlcNAc, N-acetylglucosamine; NS, N-sulfation; 6S, 6-O-sulfation; 4S, 4-O-sulfation; GalNAc, N-acetylgalactosamine; HS, heparan sulfate; CS, chondroitin sulfate; MIS-C, multisystem inflammatory syndrome in children; UA, uronic acid

Since glycosaminoglycan sulfation content may vary in inflammatory conditions [35], we also performed a structural analysis on glycosaminoglycan fragments. As detailed in Fig. 3, heparan sulfate disaccharides from healthy controls were mostly non- and monosulfated, with minor presence of highly sulfated ones, which increased notably in shock patients. Consequently, higher degree of sulfation (ds, expressed as number of sulfates/disaccharide) could be observed in MIS-C patients presenting with shock (ds = 1.6) as compared to non-shock patients (ds = 1) or healthy controls (ds = 0.9). Completely different profiles were observed for enoxaparin and heparin, mainly constituted by trisulfated (~ 67%) and disulfated disaccharides (~ 17%) with consequent high ds (2.5). The analysis of chondroitin sulfate structure demonstrated the presence of mainly 4-O-sulfated disaccharides in all samples with similar ds between healthy, non-shock, and shock patients (ds = 0.9 vs 0.8 vs 1, respectively).

## Discussion

In this study, we found that MIS-C patients presented significant damage to the vascular glycocalyx as characterized by important syndecan-1 shedding. The extent of glycocalyx shedding was associated with disease severity, in particular hemodynamic instability, and correlated with TNF-α concentration.

Recent paraclinical and clinical studies have suggested that injury to the endothelium causing endothelial dysfunction may play a major role in COVID-19 pathogenesis and complications [36, 37]. An autopsy report from patients who died from COVID-19 found evidence of severe endothelial injury associated with intracellular SARS-CoV-2 virus inclusion causing endotheliitis in lung tissue [36]. The degree of coagulopathy in patients with severe COVID-19 characterized by increased d-dimer, fibrinogen degradation levels, and prolonged prothrombin time was associated with poor prognosis [38]. In line with our results, glycocalyx shedding has been also recently reported in adult patients with COVID-19 who presented elevated plasma or serum syndecan-1 levels, as compared to healthy subjects or non-COVID-19 ICU patients, that remained elevated up to 7 days after admission [21–24, 39, 40]. The integrity of the glycocalyx is essential to maintain vascular homeostasis [41]; accordingly, dysfunction may contribute to tissue edema, aberrant vascular tone, and inappropriate inflammation, which are all characteristics of COVID-19 complications in adults but also of MIS-C clinical presentation in children.

A significant proportion of children with MIS-C present clinical signs and symptoms overlapping with Kawasaki disease (KD), an acute systemic vasculitis affecting infants and small children. Although the exact mechanism for both MIS-C and KD remains unknown, it is hypothesized that they both represent a post-infectious inflammatory response affecting genetically susceptible children complicated by coronary artery abnormalities. Unlike KD patients, MIS-C patients tend to be older and present typically with a more pronounced proinflammatory response and signs of myocardial injury with shock. Interestingly, this clinical presentation of MIS-C is similar to a severe subtype of KD, i.e., Kawasaki disease shock syndrome, characterized by more severe proinflammatory cytokine production, IVIG non-responsiveness, and coronary artery abnormalities [42]. The utility of glycocalyx shedding markers in KD patients was recently evaluated by Ohnishi et al. [43]. They reported that circulating syndecan-1 and hyaluronan were elevated in children with KD as compared to febrile and non-febrile control children. Interestingly, children who developed coronary artery abnormalities presented significantly higher syndecan-1 and hyaluronan concentration during the acute phase before IVIG administration, as compared to KD children who did not develop coronary artery abnormalities. In our cohort of patients, three patients developed coronary artery dilatations with very elevated syndecan-1 concentration in one patient (1600 ng/mL) and moderately elevated syndecan-1 in the two other patients (100 ng/mL and 284.5 ng/mL). The clinical utility of serum glycocalyx components as biomarkers to predict onset of coronary artery abnormalities will need to be evaluated in future larger clinical studies.

Heparan sulfate structure is remodeled by sulfatases and heparanase that change the sulfation and length of heparan sulfate chains on cell-surface syndecans and consequently of circulating fragments [44], therefore impacting both short-distance and long-distance interactions. For example, highly sulfated heparan sulfate fragments that circulate and interact with components of the bloodstream could contribute to the progression of the inflammation and consequent glycocalyx degradation. Indeed, heparan sulfate in serum from septic shock patients has been shown to induce mitochondrial dysfunction in cardiomyocytes in a Toll-like receptor 4 dependent manner [45]. Further investigation of the time course levels of glycosaminoglycans in MIS-C patients, together with other parameters (sulfatase, heparanase), would probably be necessary to confirm this hypothesis. Together with increased syndecan-1 levels in plasma, we observed significant increase of heparan sulfate in urine in MIS-C patients with shock, specifically sulfated fragments. A consideration that must be done is that while syndecan-1 can be easily detected by ELISA, the isolation of urinary heparan sulfate, followed by depolymerization and structural analysis, is delicate in patients undergoing intravenous UFH treatment. Although aPTT was not increased to anticoagulated level in our patients, clearance of UFH could have contributed to augmented disaccharides observed in patient MISC05, while this is not the case for patients treated with enoxaparin. Indeed, the urinary excretion of enoxaparin represents around 8.7% of the subcutaneous administered dose [46], with negligible plasma anti-IIa activity 12 h post-subcutaneous injection and little anti-Xa activity, as reported by Fareed et al. [47] and Frydman et al. [48].

Numerous preclinical and clinical studies have shown that proinflammatory cytokines and excessive reactive oxygen species represent the main actors in glycocalyx degradation in sepsis via activation of proteinases and glycosidases [49, 50]. Interestingly, animal models of sepsis have demonstrated that glycocalyx degradation involved the activation of endothelial heparanase, via TNF-α-dependent mechanisms [51]. Both severe COVID-19 and MIS-C are characterized by excessive or uncontrolled release of cytokines, suggesting the development of a cytokine storm triggered by viral infection [52]. Two recent studies have shown the implication of heparanase in glycocalyx shedding in adults with COVID-19 [21, 24]. Furthermore, a recent inflammation profiling of COVID-19 patients reports the presence of a unique inflammatory profile in COVID-19 patients as compared to non-COVID-19 ICU patients characterized notably by early and sustained elevations in circulating TNF-α, granzyme B, and elastase 2 [53]. These data suggest that the mechanism of glycocalyx degradation in COVID-19 may be similar to sepsis through heparanase activation. Whether it is also the case in MIS-C remains to be determined.

The current management of MIS-C is extrapolated from Kawasaki disease and focus on restoration of immune homeostasis with IVIG, corticosteroids, and immunomodulators and the prevention of vascular complications with antiplatelet therapy and anticoagulation. Recent evidence from COVID-19 literature [21–24, 39, 40], supported by our findings, raises the question whether the preservation and restoration of the glycocalyx should not be part of the therapeutic goals for those patients. It has been shown in both preclinical and clinical studies that hydrocortisone and heparin infusion could protect the glycocalyx from degradation during ischemia/reperfusion [54] and sepsis [49, 51]. Interestingly, heparin’s protective effect was mediated by blocking TNF- α activation of endothelial heparanase activity. We may therefore speculate that the positive response of MIS-C patients treated with IVIG and corticosteroids associated with antiplatelet therapy and prophylactic anticoagulation with heparin [55] may involve the preservation and restoration of glycocalyx in these patients. Our data showed that decrease in glycocalyx shedding corresponded to clinical improvement, with syndecan-1 value normalizing over time in all patients.

Hypervolemia has been associated with increased glycocalyx degradation in sepsis [56] and preclinical studies suggest that hypervolemia induces the release of atrial natriuretic peptide in response to mechanical wall stress, which in turn degrade the glycocalyx [57]. In our study, patients presenting with a more severe form of MIS-C received larger volume of fluid resuscitation at the emergency room and developed earlier and more pronounced fluid overload with very elevated NT-proBNP levels as compared to patients with a moderate form of MIS-C. Since hypervolemia may exacerbate glycocalyx degradation, children with severe MIS-C may be particularly more susceptible to fluid resuscitation and therapies with large volume such as IVIG.

There are some limitations to this study. First, it was an observational exploratory study from a small patient cohort restricted to 17 MIS-C patients and 5 healthy controls and therefore sensitive to confounders and selection bias, thus limiting the generalization of our findings. However, age, sex, and clinical presentation from this patient cohort are comparable to larger observation MIS-C cohort [5] and the sample size was similar to most other exploratory MIS-C studies. Our results identified syndecan-1 as a potential useful biomarker to evaluate the severity of vascular injury and help in the identification of high-risk patients with MIS-C who require more aggressive treatment and monitoring. Secondly, this study was neither designed nor powered to test the performance of glycocalyx markers for outcome prediction. Thirdly, all the blood and urine analysis of glycocalyx function were performed on samples collected as parts of clinical routine without predetermined timepoints, which precluded extended correlation analysis using the different glycocalyx markers. Therefore, these data should be regarded as exploratory and hypothesis-generating clearly deserving validation in larger, prospective studies with serial blood drawings.

## Conclusion

Our data suggest that syndecan-1 level correlating with increased soluble heparan sulfate and chondroitin sulfate reflects the severity of endothelial injury and represents a potential useful biomarker in patients with MIS-C. Whether glycocalyx damage plays a role in MIS-C pathophysiology or is simply a marker of illness severity remains to be clarified. Through its central role to maintain vascular homeostasis, endothelial glycocalyx represents an attractive therapeutic target for patients with severe COVID-19 or MIS-C.

## Supplementary information

Below is the link to the electronic supplementary material.

Supplemental Figure 1. Augmentation of syndecan-1 in MIS-C patients over time, expressed as ratio on control. (a) Admission level of plasma and serum syndecan-1 expressed as ratio on the relative control. Time course of syndecan-1 plasma concentration since hospital admission is reported in MIS-C patients with shock (b) or without shock (C). Data are presented as median with interquartile range. *P < 0.05, Mann Whitney test. MIS-C = multisystem inflammatory syndrome in children; ctrl = control. (EPS 155 KB)

Supplemental Figure 2. Sulfation pattern of urinary heparan sulfate and chondroitin sulfate from children with MIS-C. The percentages of variously substituted disaccharides are reported for HS (a) and for CS (c). The overall sulfation degree (sulfates/disaccharide) is shown for HS (b) and for CS (d). Analysis of two samples of enoxaparin is reported together with HS and expressed as median. Data from healthy controls are expressed as median with interquartile range. ΔUA = unsaturated uronic acid ± 2-O-sulfation (2S); GlcNAc = N-acetylglucosamine; NS= Nsulfation; 6S=6-O-sulfation; 4S=4-O-sulfation; GalNAc= N-acetylgalactosamine. HS = heparan sulfate; CS = chondroitin sulfate; MIS-C = multisystem inflammatory syndrome in children; UA = uronic acid. (EPS 171 KB)

Supplemental Figure 3. Quantification of urinary HS through RPIP-HPLC in MIS-C patients after correction for heparin residues. The figure reports the urinary heparan sulfate (µg/mg creatinine) concentration in MIS-C patients. Heparan sulfate concentration in the MIS-C shock group is reported with and without subtracting heparin contribution for MISC05 patient who was treated with continuous intravenous unfractionated heparin at the time of urinary analysis. Data are expressed as median with interquartile range. *P < 0.05, Kruskal-Wallis test with FDR correction. HS = heparan sulfate; MIS-C = multisystem inflammatory syndrome in children; RPIP-HPLC = reverse-phase ion-pairing high-performance liquid chromatography. (EPS 132 KB)

Supplementary file4 (DOCX 20 KB)

## Data Availability

The datasets generated during and/or analyzed during the current study are available from the corresponding author on reasonable request.
