# Supplementary material for: Endothelial glycocalyx degradation in multisystem inflammatory syndrome in children related to COVID-19
Source: J Mol Med (Berl). 2022 Mar 26;100(5):735–46. doi: 10.1007/s00109-022-02190-7 (PMC8960079; doi:10.1007/s00109-022-02190-7)
Supplement: Supplementary file 4 — Supplementary file4 (DOCX 20 KB) [file 109_2022_2190_MOESM4_ESM.docx]

| **Supplementary Table 1: Demographics, clinical findings, imaging findings, treatments, and outcome** | | | | | | | | | | | |
| --- | --- | --- | --- | --- | --- | --- | --- | --- | --- | --- | --- |
|  | **Age; weight;**  **BMI; comorbidities** | **Clinical presentation** | |  | **Number of organ dysfunction** | **Organ support** | **Pharmacological treatment** | **Imaging results** | **Echocardiography** | **Microbiology results** | **PICU and hospital length of stay; outcome** |
|  |  | Initial | At admission | PICU referral |  |  |  |  |  |  |  |
| MISC1 (male, Asian) | 10 years; 52 kg; BMI 25.9 kg/m^2^; no comorbidities | 6 days fever > 39°C; headaches; dry cough; abdominal pain; vomiting; conjunctivitis | Day 6 of illness; BP 86/44 mmHg; HR 131 beats/min; RR 36 breaths/min; work of breathing; SatO_2_ 96% RA | Yes | 4 | NIV | Noradrenaline, hydroxychloroquine, azithromycin, heparin, piperacillin/ tazobactam | CT: Right lobar consolidation with fluid overload; ileo-colitis and acute mesenteric lymphadenitis | Normal biventricular function; no coronary artery abnormalities | SARS-CoV-2 positive (negative PCR but positive serological testing with IgG and IgA antibodies); sterile blood cultures | 7 days in PICU; 12 days total at the hospital; alive |
| MISC2 (male, African) | 10 years; 55 kg; BMI 29 kg/m^2^; no comorbidities | 7 days fever > 39°C; abdominal pain; vomiting | Day 7 of illness; BP 85/51 mmHg; HR 115 beats/min; RR 39 breaths/min; SatO_2_ 98% RA | Yes | 5 | MV, RRT | Noradrenaline, vasopressin, hydroxychloroquine, azithromycin, hydrocortisone, anakinra, IVIG, , meropenem, vancomycin, caspofungin, heparin, ASA | CT: Bilateral basal lung consolidations; acute mesenteric lymphadenitis | LV dysfunction; presence of coronary artery abnormalities | SARS-CoV-2 positive (negative PCR but positive serological testing with IgG and IgA antibodies); sterile blood cultures | 14 days in PICU; 19 days total at the hospital; alive |
| MISC3 (male, Caucasian) | 13 years; 65 kg; BMI 18.6 kg/m^2^; no comorbidities | 8 days fever > 39°C; abdominal pain; odynophagia; rash | Day 8 of illness; BP 101/68 mmHg; HR 116 beats/min; RR 17 breaths/min; SatO_2_ 98% RA | No | 1 | None | Azithromycin, ceftriaxone, heparin, ASA | CT: Bilateral basal lung consolidations with pleural effusion; acute mesenteric lymphadenitis | Normal biventricular function; no coronary artery abnormalities | SARS-CoV-2 positive (negative PCR but positive serological testing with IgG and IgA antibodies); sterile blood cultures | 8 days total at the hospital; alive |
| MISC4 (male, Hispanic) | 10 years; 32 kg; BMI 14.8 kg/m^2^; no comorbidities | 3 days fever > 39°C; abdominal pain | Day 3 of illness; BP 121/76 mmHg; HR 145 beats/min; RR 20 breaths/min; SatO_2_ 100% RA | No | 0 | None | Hydroxychloroquine, tocilizumab, ceftriaxone, metronidazole, heparin, ASA | CT: Ileo-colitis and acute mesenteric lymphadenitis and a splenic hemangioma | Normal biventricular function; no coronary artery abnormalities | SARS-CoV-2 positive (negative PCR but positive serological testing with IgG and IgA antibodies); adenovirus positive in stools; sterile blood cultures | 9 days total at the hospital; alive |
| MISC5 (male, African) | 11 years; 39 kg; BMI 17.3 kg/m^2^; no comorbidities | 7 days of fever >39°C; headaches; diarrhea and vomiting; rash and bilateral conjunctivitis | Day 8 of illness; BP 77/48 mmHg; HR 146 beats/min; RR 43 breaths/min; SatO_2_ 100% RA | Yes | 5 | MV | Noradrenaline, milrinone, adrenaline, vasopressine , hydroxychloroquine, hydrocortisone, methylprednisolone, anakinra (anti-IL-1), tocilizumab; IVIG, ceftriaxone, heparin, ASA | CT: Bilateral basal lung consolidations with pleural effusion; ileo-colitis and acute mesenteric lymphadenitis | LV dysfunction; no coronary artery abnormalities | SARS-CoV-2 positive (negative PCR but positive serological testing with IgG and IgA antibodies); sterile blood cultures | 11 days in PICU; 40 days total at the hospital; alive |
| MISC6 (female, mixed Caucasian/Asian) | 11 years; 29.8 kg; BMI 16.1 kg/m^2^; no comorbidities | 3 days of fever > 39°C; abdominal pain; rash; conjunctivitis; cheilitis | Day 4 of illness; BP 110/67 mmHg; HR 136 beats/min; RR 15 breaths/min; SatO_2_ 98% RA | Yes | 3 | None | Noradrenaline, methylprednisolone, IVIG, tocilizumab, anakinra, ceftriaxone, meropenem, vancomycin, heparin, ASA | Normal CXR | Normal biventricular function; no coronary artery abnormalities | SARS-CoV-2 positive (negative PCR but positive serological testing with IgG and IgA antibodies); sterile blood cultures | 9 days in PICU; 10 days total at the hospital; alive |
| MISC7 (male, Caucasian) | 15 years; 68 kg; BMI 20.3 kg/m^2^; no comorbidities | 4 days of fever > 39°C; odynophagia; diarrhea; vomiting | Day 5 of illness; BP 80/46 mmHg; HR 128 beats/min; RR 33 breaths/min; SatO_2_ 98% RA | Yes | 5 | None | Noradrenaline, milrinone, methylprednisolone, IVIG, anakinra, ceftriaxone, heparin, ASA | Normal CXR | Biventricular dysfunction; presence of coronary artery abnormalities | SARS-CoV-2 positive (negative PCR but positive serological testing with IgG and IgA antibodies); sterile blood cultures | 7 days in PICU; 10 days total at the hospital; alive |
| MISC8 (male, mixed Caucasian/African) | 13 years; 46kg; BMI 19.9 kg/m^2^; no comorbidities | 5 days of fever > 39°C; abdominal pain; diarrhea; vomiting | Day 7 of illness; BP 70/41 mmHg; HR 120 beats/min; RR 40 breaths/min; SatO_2_ 94% RA | Yes | 3 | None | Noradrenaline, methylprednisolone, IVIG, anakinra, ceftriaxone, heparin, ASA | Normal CXR | Normal biventricular function; no coronary artery abnormalities | SARS-CoV-2 positive (negative PCR but positive serological testing with IgG and IgA antibodies); sterile blood cultures | 6 days in PICU; 12 days total at the hospital; alive |
| MISC9 (male, Caucasian) | 13 years; 39.5kg; BMI 14.9 kg/m^2^; no comorbidities | 7 days of fever > 39°C; dry cough; loss of appetite | Day 8 of illness; BP 99/73 mmHg; HR 112 beats/min; RR 22 breaths/min; SatO_2_ 97% RA | Yes | 0 | None | Methylprednisolone, IVIG, ceftriaxone, heparin, ASA | Normal CXR; abdominal ultrasound: acute mesenteric lymphadenitis | LV dysfunction; no coronary artery abnormalities | SARS-CoV-2 positive (negative PCR but positive serological testing with IgG and IgA antibodies); sterile blood cultures | 2 days in PICU; 5 days total at the hospital; alive |
| MISC10 (male, mixed Caucasian/African) | 2 years; 14.5kg; no comorbidities | 3 days of fever > 39°C; irritability; rash; conjunctivitis | Day 6 of illness; BP 90/54 mmHg; HR 136 beats/min; RR 45 breaths/min; SatO_2_ 100% on 5L/min oxygen | Yes | 2 | None | Noradrenaline, methylprednisolone, IVIG, ceftriaxone, heparin, ASA | No imaging | Normal biventricular function; no coronary artery abnormalities | SARS-CoV-2 positive (negative PCR but positive serological testing with IgG and IgA antibodies); sterile blood cultures | 2 days in PICU; 7 days total at the hospital; alive |
| MISC12 (male, Caucasian) | 7 years; 50kg; BMI 23.8 kg/m^2^; no comorbidities | 4 days of fever > 39°C; abdominal pain; vomiting; rash | Day 5 of illness; BP 87/47 mmHg; HR 125 beats/min; RR 41 breaths/min; SatO_2_ 100% on 5L/min oxygen | Yes | 3 | None | Noradrenaline, methylprednisolone, IVIG, ceftriaxone, heparin, ASA | CT: Bilateral basal lung consolidations; acute mesenteric lymphadenitis | LV dysfunction; no coronary artery abnormalities | SARS-CoV-2 positive (negative PCR but positive serological testing with IgG and IgA antibodies); sterile blood cultures | 4 days in PICU; 7 days total at the hospital; alive |
| MISC13 (female, mixed Caucasian/Asian) | 8 years; 28.5kg; BMI 14.3 kg/m^2^; no comorbidities | 5 days of fever > 39°C, headaches; asthenia; abdominal pain; vomiting; rash; conjunctivitis | Day 6 of illness; BP 75/42 mmHg; HR 111 beats/min; RR 26 breaths/min; SatO_2_ 96% RA | Yes | 3 | None | Noradrenaline, methylprednisolone, IVIG, ceftriaxone, heparin, ASA | Normal CXR | Normal biventricular function; no coronary artery abnormalities | SARS-CoV-2 positive (negative PCR but positive serological testing with IgG and IgA antibodies); sterile blood cultures | 2 days in PICU; 7 days total at the hospital; alive |
| MISC14 (male, mixed Caucasian/African) | 5 years; 18kg; BMI 14.1 kg/m^2^; no comorbidities | 5 days of fever > 39°C; abdominal pain; loss of appetite; rash; conjunctivitis | Day 6 of illness; BP 83/59 mmHg; HR 118 beats/min; RR 30 breaths/min; SatO_2_ 100% on RA | Yes | 0 | None | Methylprednisolone, IVIG, ceftriaxone, heparin, ASA | Normal CXR | Normal biventricular function; no coronary artery abnormalities | SARS-CoV-2 positive (negative PCR but positive serological testing with IgG and IgA antibodies); sterile blood cultures | 1 day in PICU; 6 days total at the hospital; alive |
| MISC15 (male, Hispanic) | 13 years; 77.6kg; BMI 25.2 kg/m^2^; no comorbidities | 2 days of fever > 39°C; headaches; cough; rhinorrhea; abdominal pain; vomiting | Day 4 of illness; BP 123/72 mmHg; HR 78 beats/min; RR 16 breaths/min; SatO_2_ 98% RA | Yes | 0 | None | Methylprednisolone, IVIG, ceftriaxone, heparin, ASA | CT: ileo-colitis and acute mesenteric lymphadenitis | Normal biventricular function; no coronary artery abnormalities | SARS-CoV-2 positive (negative PCR but positive serological testing with IgG and IgA antibodies); sterile blood cultures | 1 day in PICU; 7 days total at the hospital; alive |
| MISC16 (male, Caucasian) | 8 years; 26kg; BMI 13.9 kg/m^2^; no comorbidities | 2 days of fever > 39°C; vomiting; | Day 2 of illness; BP 104/57 mmHg; HR 124 beats/min; RR 22 breaths/min; SatO_2_ 99% RA | Yes | 1 | None | Methylprednisolone, IVIG, ceftriaxone, heparin, ASA | Normal CXR; abdominal ultrasound: acute mesenteric lymphadenitis | LV dysfunction; presence of coronary artery abnormalities | SARS-CoV-2 positive (negative PCR but positive serological testing with IgG and IgA antibodies); sterile blood cultures | 5 days in PICU; 6 days total at the hospital; alive |
| MISC17 (male, Caucasian) | 15 years; 54kg; BMI 18 kg/m^2^; no comorbidities | 6 days of fever > 39°C; headaches; myalgia; diarrhea; vomiting; conjunctivitis; rash | Day 6 of illness; BP 95/56 mmHg; HR 75 beats/min; RR 18 breaths/min; SatO_2_ 99% RA | Yes | 0 | None | Methylprednisolone, IVIG, ceftriaxone, heparin, ASA | No exam | Normal biventricular function; no coronary artery abnormalities | SARS-CoV-2 positive (negative PCR but positive serological testing with IgG and IgA antibodies); sterile blood cultures | 1 day in PICU; 5 days total at the hospital; alive |
| MISC18 (male, African) | 13 years; 92.2kg; BMI 28 kg/m^2^; no comorbidities | 5 days of fever > 39°C; headaches; cough; vomiting; | Day 6 of illness; BP 108/45 mmHg; HR 109 beats/min; RR 30 breaths/min; SatO_2_ 100% RA | Yes | 2 | None | Noradrenaline, methylprednisolone, IVIG, ceftriaxone, clindamycin, heparin, ASA | Normal CXR | Normal biventricular function; no coronary artery abnormalities | SARS-CoV-2 positive (negative PCR but positive serological testing with IgG and IgA antibodies); sterile blood cultures | 5 days in PICU; 7 days total at the hospital; alive |
| ASA = acetylsalicylic acid; BMI= body mass index; BP= blood pressure; COVID-19= coronavirus disease 2019; CT= computed tomography; CXR= chest x-ray; FiO_2_ = fraction of inspired oxygen; HR = heart rate; IVIG = human intravenous immunoglobulin; LV = left ventricle; MV = mechanical ventilation via endotracheal tube; NIV = non-invasive ventilation; NP = not performed; PCR = polymerase chain reaction; PICU = pediatric intensive care unit; RA = room air; RR = respiratory rate; RRT = renal replacement therapy; SARS-CoV-2 = severe acute respiratory syndrome coronavirus 2; SatO_2_ = oxygen saturation; | | | | | | | | | | | |
